# A diagnostic accuracy study comparing RNA LAMP, direct LAMP, and rapid antigen testing from nasopharyngeal swabs

**DOI:** 10.3389/fmicb.2022.1063414

**Published:** 2022-12-22

**Authors:** Guojun Cao, Ke Lin, Jingwen Ai, Jianpeng Cai, Haocheng Zhang, Yiqi Yu, Qihui Liu, Xinyun Zhang, Yi Zhang, Zhangfan Fu, Jieyu Song, Hongyu Wang, Guanmin Yuan, Sen Wang, Ming Guan, Wenhong Zhang

**Affiliations:** ^1^Department of Laboratory Medicine, Huashan Hospital, Shanghai Medical College, Fudan University, Shanghai, China; ^2^Department of Infectious Diseases, National Medical Center for Infectious Diseases, Shanghai Key Laboratory of Infectious Diseases and Biosafety Emergency Response, Huashan Hospital, Fudan University, Shanghai, China; ^3^Shanghai Huashan Institute of Microbes and Infections, Shanghai, China; ^4^National Clinical Research Center for Aging and Medicine, Huashan Hospital, Fudan University, Shanghai, China

**Keywords:** SARS-CoV-2, COVID-19, LAMP assay, rapid antigen test, diagnostic accuracy

## Abstract

**Introduction:**

During the coronavirus disease 2019 (COVID-19) pandemic, the early detection and isolation of individuals infected with severe acute respiratory syndrome coronavirus disease 2 (SARS-CoV-2) through mass testing can effectively prevent disease transmission. SARS-CoV-2 nucleic acid rapid detection based on loop-mediated isothermal amplification (LAMP) may be appropriate to include in testing procedures.

**Methods:**

We used 860 nasopharyngeal specimens from healthcare workers of Huashan Hospital and COVID-19 patients collected from April 7th to 21st, 2022, to assess the clinical diagnostic performance of the LAMP assay marketed by Shanghai GeneSc Biotech and compared it to the result of a rapid antigen test (RAT) head-to-head.

**Results:**

Overall, the diagnostic performance of LAMP assay and RAT were as follows. The LAMP assay represented higher sensitivity and specificity than RAT, especially in the extracted RNA samples. The sensitivity was 70.92% and 92.91% for direct LAMP and RNA-LAMP assay, respectively, while the specificity was 99.86% and 98.33%. The LAMP assay had overall better diagnostic performance on the specimens with relatively lower *C*_t_ values or collected in the early phase (≤7 days) of COVID-19. The combination of LAMP assay and RAT improved diagnostic efficiency, providing new strategies for rapidly detecting SARS-CoV-2.

**Conclusion:**

The LAMP assay are suitable for mass screenings of SARS-CoV-2 infections in the general population.

## Introduction

The pandemic caused by coronavirus disease 2019 (COVID-19), which began in December 2019 in Wuhan, China, results in a global public health crisis (Andersen et al., 2020). This disease is caused by infection with severe acute respiratory syndrome coronavirus 2 (SARS-CoV-2), a highly-transmissible virus belonging to the *Coronaviridae* family, of which, the control remains a global crisis (Andersen et al., 2020). The World Health Organization (WHO) declared a global emergency over the novel coronavirus on January 30, 2020 and declared COVID-19 a pandemic disease on March 12, 2020 (Cucinotta and Vanelli, 2020). According to WHO records, as of May 10, 2022, over 0.5 billion cases and over 6 million deaths had been reported (WHO, 2022). Rapid diagnosis is essential for screening SARS-CoV-2 infections and is important for controlling its spread.

Current methods for COVID-19 testing mainly fall into three categories: nucleic acid tests, serological tests for antibody detection, and antigen tests. Reverse-transcription polymerase chain reaction (RT-PCR), the gold standard for COVID-19 diagnosis, has the advantages of high sensitivity and specificity, and has become the most widely used technology for amplifying nucleic acids. However, RT-PCR testing has many limitations, including its long detection time, costly instruments, and need for skilled operators. As a result of these concerns, rapid antigen tests (RATs) for COVID-19, which can be easily performed and interpreted without complex equipment, have been approved for clinical use worldwide. However, studies have shown that RATs can only confirm samples containing large amounts of the virus as positive cases, while those containing small amounts of the virus may be misinterpreted, thus some infected patients may be missed (Yamayoshi et al., 2020). These limitations have prompted researchers to search for a method providing rapid and accurate detection of SARS-CoV-2.

The loop-mediated isothermal amplification (LAMP) technique was developed by Notomi et al. (2000). It is a rapid and straightforward technique for nucleic acid detection with high specificity and sensitivity. The specific amplification of the LAMP method relies on 4 or 6 designed primers that bind to six regions specific to the target gene (Notomi et al., 2000; Enosawa et al., 2003). Compared to RT-PCR, LAMP assays are significantly more rapid, with the advantages of no temperature changes, simpler operation, less cost, and more straightforward result interpretation (Enosawa et al., 2003; Chen et al., 2015). With the development and improvement of LAMP technology, it is now widely applied in many fields, such as food or environmental microbiology detection, and clinical pathology diagnosis. In healthcare, LAMP is used for detecting the Zika virus, Ebola virus, yellow fever virus, SARS-CoV-2, and various infectious diseases (Kwallah et al., 2013; Li et al., 2015; Chotiwan et al., 2017; Kashir and Yaqinuddin, 2020). Meanwhile, LAMP technology could be compatible with pH-based colorimetric readings (Tanner et al., 2015), allowing straightforward interpretation of test results by visual inspection. Considering its various advantages, LAMP is appealing in point-of-care or low-resource settings and can meet the need for at-home self-testing. Some LAMP-based kits for SARS-CoV-2 detection have received emergency use authorization (EUA) from the US Food and Drug Administration (FDA; FDA, 2022).

Novel and precise diagnostic tools that can be applied to multiple scenarios could help contain the present epidemic caused by SARS-CoV-2. Varieties of LAMP-based kits with high sensitivity and specificity of SARS-CoV-2 examination are available today, including the Loopamp 2019-SARS-CoV-2 detection reagent kit, the Warmstart RT-LAMP Assay Kit, the Variplex RT-LAMP Assay Kit, the RT-LAMP Mastermix kit, the specific colorimetric and fluorescence RT-LAMP Kit (Baek et al., 2020; Kitagawa et al., 2020; Klein et al., 2020; Lee et al., 2020; Nagura-Ikeda et al., 2020; Rödel et al., 2020; Yan et al., 2020; Kitajima et al., 2021). In this study, we wish to explore more suitable application scenarios of the LAMP assay and estimated the sensitivities and specificity of both the LAMP assay and RAT with large sample size during the SARS-CoV-2 outbreak in March 2022. We further analyzed the efficacy of the combined use of the LAMP assay and RAT, using RT-PCR as a reference.

## Materials and methods

### Study design and study population

This single-center prospective study at Huashan Hospital was carried out during April 7–21, 2022. All participants were ≥ 18 years of age. The detailed enrollment criteria are listed in Supplementary Table S1. After enrollment and consent, participants completed an initial survey to collect information on demographics, including age, gender, and identification number. The information of individual’s health history was gathered, including the date of the first positive report of a SARS-CoV-2 RT-PCR test and diagnosed with COVID-19, and the clinical classification. Participants received ≥1 reverse-transcription LAMP (RT-LAMP) test (Shanghai GeneSc Biotech Co., Ltd., Shanghai, China) with nasopharyngeal swabs (NPSs), and participants also underwent a RAT (Guangzhou Wondfo Biotech Co., Ltd., Guangzhou, China) for SARS-CoV-2 (with NPSs; Supplementary Figure S1). This study was performed in compliance with an institutional review board protocol at Huashan Hospital (2022–539).

### RT-PCR assay

RT-PCR detection of the ORF1ab and N genes of SARS-CoV-2 was performed. The test procedure was as follows: first, purified viral nucleic acid (RNA) was extracted using the QIAamp viral RNA mini kit (50) extraction kit (article no. 52904; Qiagen, Hilden, Germany) or nucleic acid extraction rapid kit (magnetic bead methods; no. SDKF60101; Jiangsu Bioperfectus Technologies Co., Ltd., Jiangsu, China). Subsequently, an RT-PCR assay was performed after sampling, following the manufacturer’s instructions, including preparing the reaction system, adding the treated sample, and then amplification was performed on a TK-6000 real-time quantitative thermal cycler (Anhui Toneker Biotechnology Co., Ltd., Jinzhai, China) for approximately 1.5 h. Finally, we analyzed the result.

### RT-LAMP assay/novel coronavirus 2019-nCoV nucleic acid rapid detection Kit (LAMP)

**Figure 1:**
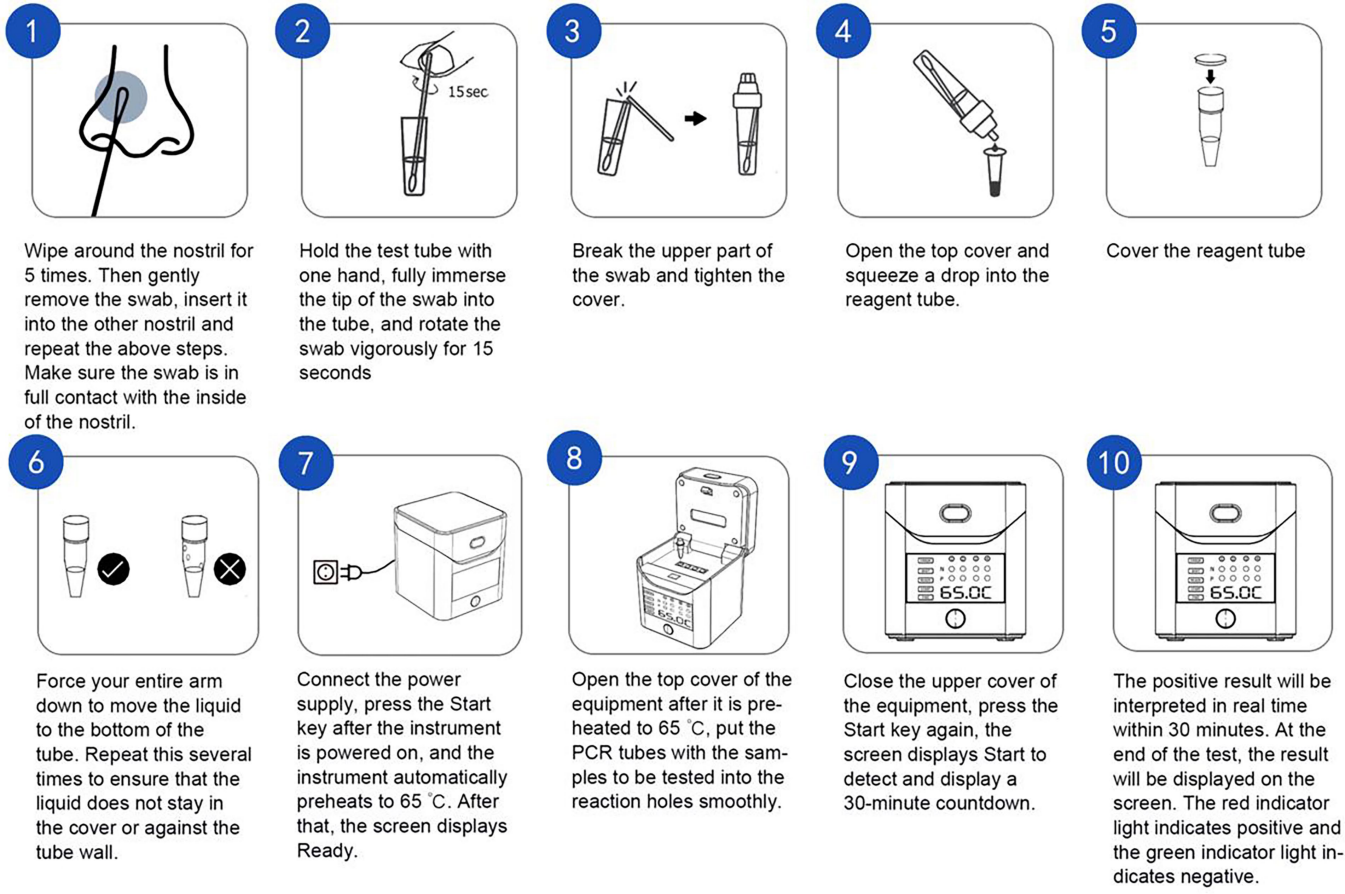
Operation flow of the LAMP assay.

NPSs were collected by experienced clinicians and placed in 2 ml of sample preservation solution provided by the manufacturer (DNase and RNase-free water); then, the samples were processed in different ways. For samples processed with RNA-LAMP assay, the purified viral nucleic acid (RNA) was extracted using a nucleic acid extraction rapid kit (magnetic bead method; no. SDKF60101; Jiangsu Bioperfectus Technologies Co., Ltd., Jiangsu, China) from the original specimen for further detection. As for the samples processed with direct LAMP assay, the original sample would be heated at 95°C for 5 min directly for further test. The RT-LAMP assay was performed immediately after sampling, following the manufacturer’s instructions. For the test procedure, we preheated the heat block to 65°C, and took out the RCOV 8-tube strip. Then, we took out 25 μl of the pre-purified nucleic acid sample or heat-treated original sample and added it into a PCR reaction tube filled with a lyophilized reagent. We capped the loaded 8-tube strip for sealing and mixing well and then centrifuged it for a short period (observing the solution periodically; the color should be red). We immediately placed the PCR reaction tube in the preheated 65°C heat block; after 30 min of reaction, we took out the PCR reaction tube, cooled the tube to room temperature, and checked for a color change. If the reagent appeared yellow, the test result was positive; if the reagent was red, the test result was negative (Figure 1).

### Rapid antigen test

NPSs were collected by experienced physicians and placed in 0.4 ml of sample extraction solution (provided by the manufacturer). An antigen assay was performed following the manufacturer’s instructions. For nasopharyngeal secretion sample extraction, the NPS was collected, inserted into the sample extraction tube solution, and rotated approximately 10 times against the inner wall of the tube. The swab head should be left in the extraction tube for 1 min, and then added 3–4 drops (around 80 μl) of the treated sample to the spiking hole of the test card. The results should be observed within 15–20 min.

### Sample collection

One NPS from a single nostril was subsequently placed in a collection tube containing RNase-free water. The order of nostrils (left or right) was randomized. For NPS, clinicians were required to insert the soft tip of the swab into the patients’ nostril until they encountered mild resistance or discomfort, then gently twist the swab against the inner wall of the nasal vestibule, and place the swab into the tube with RNase-free water. Tests were performed within 2 h of sample collection. In order to reduce interference of other factors, the same nasopharyngeal swabs were collected and tested for RAT, LAMP, and RT-qPCR assays simultaneously.

### Statistical analysis

Statistical analysis was performed using IBM SPSS Statistics (IBM Corp., Armonk, NY, USA), Microsoft Excel Version 2021 (Microsoft Corporation, Redmond, WA, USA), and VassarStats: Web Site for Statistical Computation (Richard Lowry, NY, USA). Figures were conducted by GraphPad Prism 8.0 (GraphPad Software Inc., La Jolla, CA, USA) and the ggplot2 package of R (version 3.3.5; R Foundation for Statistical Computing, Vienna, Austria).

The sensitivity and specificity of the LAMP tests and RAT were determined based on the comparison with RT-PCR results. The viral load was determined in a semiquantitative manner expressed by the number of RT-PCR cycles, and the positive test cut-off of cycle threshold (*C*_T_) was 35, as a *C*_T_ < 35 indicates a positive result. Sensitivity represents the test’s ability to identify individuals whose RT-PCR result confirms a SARS-CoV-2 infection, and specificity describes the test’s capability to identify participants with negative RT-PCR test results. The positive predictive value (PPV) and negative predictive value (NPV) describe the probability of SARS-CoV-2–infected individuals with a positive test result and no SARS-CoV-2 infection in participants with a negative test result, respectively. Accuracy indicates the test’s ability to identify healthy and infected individuals correctly, referring to the standard (RT-PCR test). The diagnostic performance of the test was calculated according to the following formulae:

Sensitivity = TP / (TP + FN).

Specificity = TN / (TN + FP).

PPV = TP / (TP + FP).

NPV = TN / (TN + FN).

Accuracy = (TP + TN) / (TP + TN + FP + FN).

The true-positive (TP) value refers to the number of positive test results that are also positive, referring to the standard, and the true-negative (TN) value represents the number of true-negative individuals identified by the method being evaluated, according to the standard. The false-positive (FP) value is the number of results that the testing method indicates are positive, but the standard indicates to be negative. The false-negative (FN) value is the number of positive results according to the standard that the testing methods reported to be negative. We also calculated 95% confidence intervals (CIs) for test performance.

For baseline characteristics, continuous variants were described by median [with interquartile range (IQR)] values, and categorical variables were expressed as numbers and percentages. The chi-squared test, Fisher’s test, and McNemar test were applied when comparing the sensitivity, specificity, PPV, NPV, and accuracy between different methods. A two-sided *p* value <0.05 was considered to be statistically significant.

## Results

### General characteristics of participants

Table 1 shows the demographic information for study participants reported here. A total of 571 individuals were enrolled, 383 (67.08%) of whom were healthcare workers of Huashan Hospital, and 188 (32.92%) patients were confirmed to have COVID-19. The median age of all participants was 44 years, and 44.48% of patients were male. Of the patients enrolled, 45.74% (86/188) had asymptomatic COVID-19, while 54.26% (102/188) were symptomatic. A total of 860 NPSs were obtained from 571 participants, and all specimens were tested by direct LAMP assay, RNA-LAMP assay, and RAT.

**Table 1:** Demographic information of participants.

Characteristic	Participants
Age, year, median (IQR)	44 (33–55.5)
Gender	
Male, *n*, (%)	254 (44.48%)
Female, *n*, (%)	317 (55.52%)
COVID-19 Infection	
Uninfected, *n*, (%)	383 (67.08%)
Infected, *n*, (%)	188 (32.92%)
Asymptomatic	86 (45.74%)
Symptomatic	102 (54.26%)

### Clinical diagnostic performance of the LAMP assay and RAT

**Figure 2:**
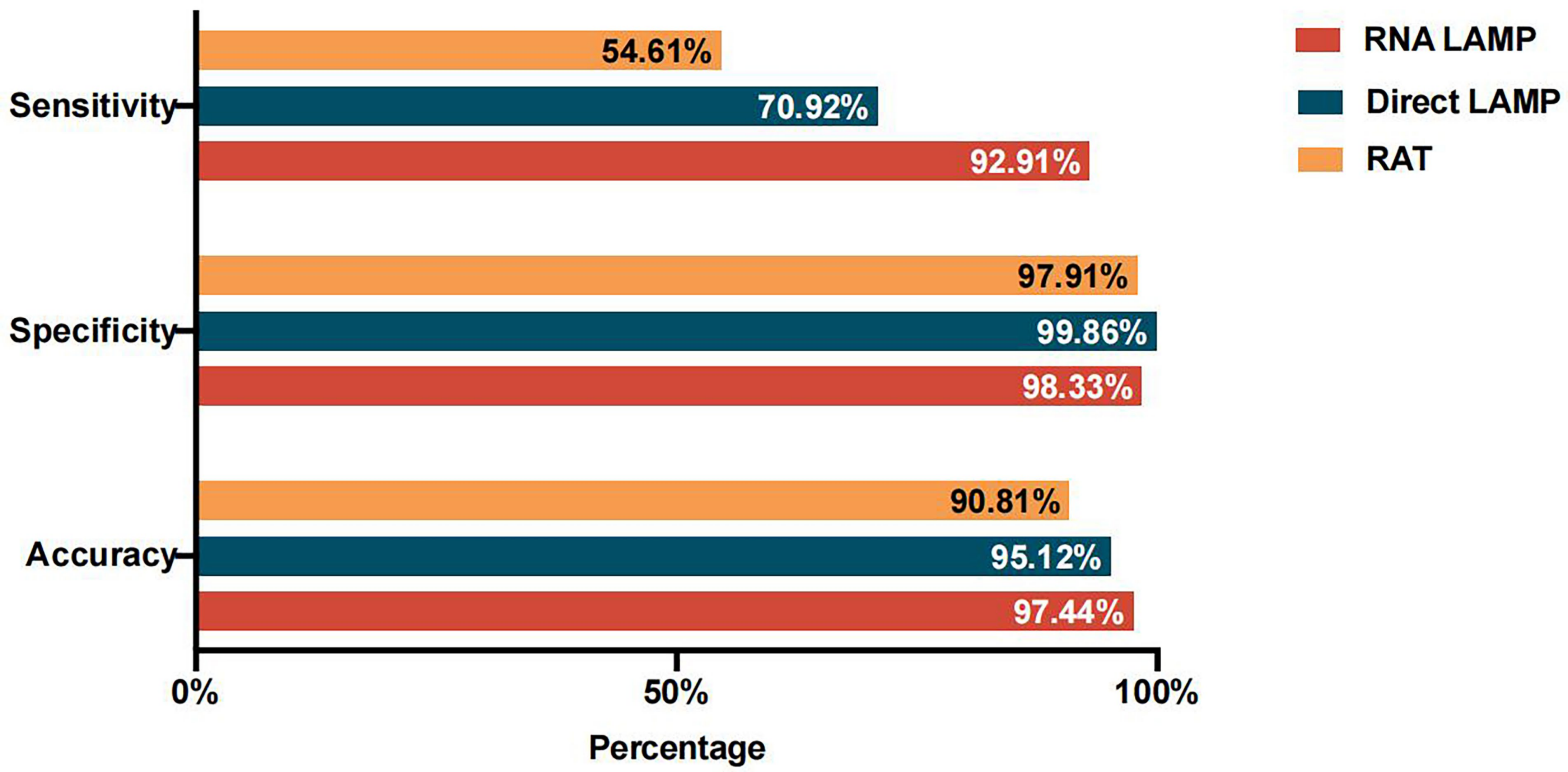
Diagnostic performance of the RNA-LAMP assay, direct LAMP assay, and RAT.

According to the clinical criteria, the specimens with the lowest *C*_T_ value ≤35 in two genes were defined as positive for SARS-CoV-2 nucleic acid. Among all the NPS samples tested, 141 specimens were positive for SARS-CoV-2 nucleic acid, while 719 were negative. Compared to the RT-PCR results, the direct LAMP assay revealed a sensitivity of 70.92% (100/141; 95% CI, 62.58%–78.10%), a specificity of 99.86% (718/719; 95% CI, 99.10%–99.99%), and an accuracy of 95.12% (818/860; 95% CI, 93.40%–96.42%). The consistency of the direct LAMP assay and RT-PCR was analyzed and the kappa value was 0.799 (*p* < 0.001), reflecting relatively good agreement. We further calculated the PPV and NPV of the direct LAMP assay, which were 99.01% (95% CI, 93.82%–99.95%) and 94.60% (95% CI, 92.68%–96.05%), respectively (Table 2; Figure 2).

**Table 2:** Clinical performance of RNA-LAMP test, LAMP test, and RAT.

	Methods			*p* Value
	RNA-LAMP	Direct LAMP	RAT	
RT-qPCR Positive (*n* = 141)	131	100	77	/
RT-qPCR Negative (*n* = 719)	707	718	704	/
Sensitivity (%) (95% CI)	92.91 (87.00–96.36)	70.92 (62.58–78.10)	54.61 (46.03–62.94)	*p*_1_ < 0.001, *p*_2_ < 0.001, *p*_3_ < 0.001
Specificity (%) (95% CI)	98.33 (97.02–99.09)	99.86 (99.10–99.99)	97.91 (96.50–98.78)	*p*_1_ = 0.001, *p*_2_ = 0.001, *p*_3_ = 0.001
PPV (%) (95% CI)	91.61 (85.49–95.39)	99.01 (93.82–99.95)	83.70 (74.21–90.29)	/
NPV (%) (95% CI)	98.61 (97.36–99.29)	94.60 (92.68–96.05)	91.67 (89.43–93.48)	/
Accuracy (%) (95% CI)	97.44 (96.09–98.35)	95.12 (93.40–96.42)	90.81 (88.63–92.62)	/
Kappa (*k*) (95% CI)	0.907 (0.888–0.926)	0.799 (0.769–0.829)	0.611 (0.572–0.650)	/
*p* Value	*p* < 0.001	*p* < 0.001	*p* < 0.001	

*p*_1_ stands for the significance of comparison between RNA-LAMP and direct LAMP.

*p*_2_ stands for the significance of comparison between RNA-LAMP and RAT.

*p*_3_ stands for the significance of comparison between direct LAMP and RAT.

In addition, the performance of the LAMP assay on extracted RNA (RNA-LAMP assay) was evaluated. Compared to the results of RT-PCR, the RNA-LAMP assay showed a sensitivity of 92.91% (131/141; 95% CI, 87.00%–96.36%), a specificity of 98.33% (707/719; 95% CI, 97.02%–99.09%), and an accuracy of 97.44% (838/860; 95% CI, 96.09%–98.35%). The consistency of RNA-LAMP assay and RT-PCR was analyzed and the kappa value was 0.907 (*p* < 0.001), reflecting great agreement. Furthermore, the PPV was 91.61% (95% CI, 85.49%–95.39%) and the NPV was 98.61% (95% CI, 97.36%–99.29%; Table 2; Figure 2).

The RAT had a sensitivity of 54.61% (77/141; 95% CI, 46.03%–62.94%), a specificity of 97.91% (704/719; 95% CI, 96.50%–98.78%), and an accuracy of 90.81% (781/860; 95% CI, 88.63%–92.62%). The consistency of RNA-LAMP assay and RT-PCR was analyzed and the kappa value was 0.611 (*p* < 0.001). Furthermore, the PPV and NPV of the RAT were 83.70% (95% CI, 74.21%–90.29%) and 91.67% (95% CI, 89.43%–93.48%), respectively (Table 2).

**Figure 3:**
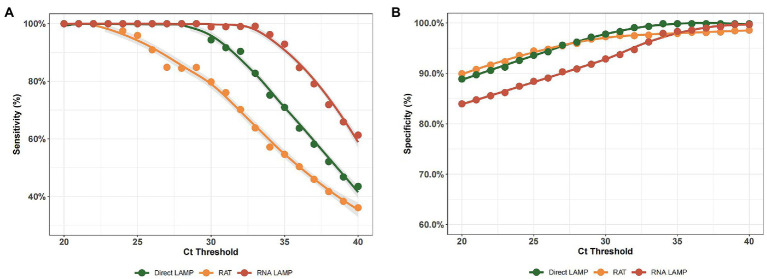
**(A)** The sensitivity of the RNA-LAMP assay, direct LAMP assay, and RAT with different RT-PCR cut-off values. **(B)** The specificity of the RNA-LAMP assay, direct LAMP assay, and RAT with different RT-PCR cut-off values.

By employing different RT-PCR cut-off values, we analyzed the variations in sensitivity and specificity of both the LAMP assay and RAT. As shown in Figures 3A,B, the LAMP assay showed better performance in identifying both positive and negative ones, while the RAT showed relatively lower sensitivity and specificity values in the *C*_T_ value range of 25–40. Additionally, direct LAMP assay achieved a similar sensitivity, regardless of whether the patient was symptomatic or not (71.64% vs. 70.27%, *p* = 0.941). Moreover, it was observed that, among both asymptomatic and symptomatic patients, direct LAMP assay represented a greater sensitivity than the RAT (asymptomatic patients: 71.64% vs. 56.72%, *p* = 0.013; symptomatic patients: 70.27% vs. 52.70%, *p* = 0.002). Direct LAMP assay also showed a higher specificity than the RAT (asymptomatic patients: 99.08% vs. 92.66%, *p* = 0.039; symptomatic patients: 100.00% vs. 94.74%, *p* = 0.016; Supplementary Table S2).

### Performance of the LAMP assay and RAT among specimens with different *C*_T_ values

Moreover, we classified patients with positive RT-PCR results into four groups according to the *C*_T_ value (Table 3). The probability of detection reflected the probability of the methods to identify the positive specimen, and it declined with an increase in *C*_T_ value in both the LAMP assay and RAT. Among patients with *C*_T_ values of 25–35, according to the RT-PCR test, the direct LAMP assay demonstrated a relatively better diagnostic performance than the RAT (25 ≤ *C*_T_ < 30: 87.50% vs. 60.00%, *p* = 0.003; 30 ≤ *C*_T_ < 35: 30.77% vs. 11.54%, *p* = 0.021). Moreover, the detection efficacy of RNA-LAMP was significantly higher than those of direct LAMP and RAT among patients with *C*_T_ values of 30–35 (82.69% vs. 30.77%, *p* < 0.001; 82.69% vs. 11.54%, *p* < 0.001).

**Table 3:** Positive detection rate of LAMP and RAT in patients with different *C*_T_ value.

*C*_T_ Value	Positive detection rate			*p* Value
	RNA-LAMP	Direct LAMP	RAT	
*C*_T_ < 20	100% (6/6)	100% (6/6)	100% (6/6)	*p*_1_, *p*_2_, *p*_3_ = NA
20 ≤ *C*_T_ < 25	100% (43/43)	100% (43/43)	95.35% (41/43)	*p*_1_ = NA, *p*_2_ = 0.5, *p*_3_ = 0.5
25 ≤ *C*_T_ < 30	97.50% (39/40)	87.50% (35/40)	60.00% (24/40)	*p*_1_ = 0.125, *p*_2_ < 0.001, *p*_3_ = 0.003
30 ≤ *C*_T_ < 35	82.69% (43/52)	30.77% (16/52)	11.54% (10/52)	*p*_1_ < 0.001, *p*_2_ < 0.001, *p*_3_ = 0.021

*p*_1_ stands for the significance of comparison between RNA-LAMP and direct LAMP.

*p*_2_ stands for the significance of comparison between RNA-LAMP and RAT.

*p*_3_ stands for the significance of comparison between direct LAMP and RAT.

NA, Not applicable.

### Effect of sampling time-point after disease onset on test sensitivity

**Figure 4:**
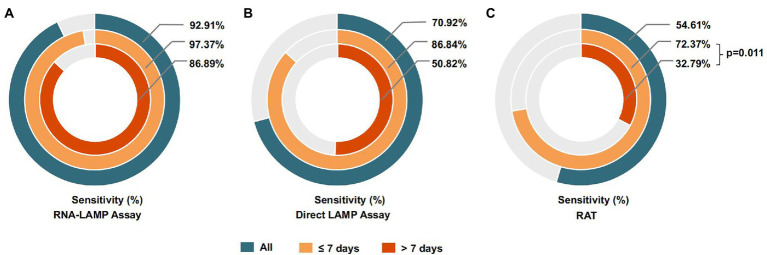
**(A)** The sensitivity of the RNA-LAMP assay among samples collected in different phases of COVID-19. **(B)** The sensitivity of the direct LAMP assay among samples collected in different phases of COVID-19. **(C)** The sensitivity of the RAT among samples collected in different phases of COVID-19.

In addition, we compared the detection effectiveness of the LAMP assay and RAT when identifying specimens collected within or over 7 days after the first positive RT-PCR results obtained from the patient. As shown in Figure 4, the direct LAMP assay provided a higher sensitivity when detecting the samples collected within 1 week from the first positive RT-PCR results, compared to those with positive results exceeding this period (86.84% vs. 50.82%, *p* = 0.053). Although the RAT also showed a higher sensitivity among patients in the early phase (≤7 days) than the late phase (>7 days; 72.37% vs. 32.79%, *p* = 0.011), it was relatively lower than that of direct LAMP (86.84% vs. 72.37%, *p* = 0.003; Table 4).

**Table 4:** Performance of LAMP and RAT at the indicated time of collection since first positive RT-qPCR results.

Methods	Early phase (≤ 7 days; *n* = 104)		Late phase (> 7 Days) (n = 282)		*p* Value
	Sensitivity (%) (95% CI)	Specificity (%) (95% CI)	Sensitivity (%) (95% CI)	Specificity (%) (95% CI)	
RNA-LAMP	97.37 (89.95–99.54)	95.65 (76.03–99.77)	86.89 (75.23–93.77)	94.98 (90.95–97.34)	*p*_4_ = 0.647
Direct LAMP	86.84 (76.68–93.17)	100.00 (82.19–100.00)	50.82 (37.83–63.71)	99.54 (97.09–99.98)	*p*_4_ = 0.053
RAT	72.37 (60.73–81.72)	95.65 (76.03–99.77)	32.79 (21.63–46.12)	93.61 (89.28–96.33)	*p*_4_ = 0.011
*p* Value	*p*_1_ = 0.008, *p*_2_ < 0.001, *p*_3_ = 0.003	*p*_1_ = 1.000, *p*_2_ = 1.000, *p*_3_ = 1.000	*p*_1_ < 0.001, *p*_2_ < 0.001, *p*_3_ = 0.013	*p*_1_ = 0.002, *p*_2_ = 0.678, *p*_3_ = 0.001	/

*p*_1_ stands for the significance of comparison between RNA-LAMP and direct LAMP.

*p*_2_ stands for the significance of comparison between RNA-LAMP and RAT.

*p*_3_ stands for the significance of comparison between direct LAMP and RAT.

*p*_4_ stands for the significance of comparison of sensitivity between the early phase and the late phase.

### Combined use of the direct LAMP assay and RAT

**Figure 5:**
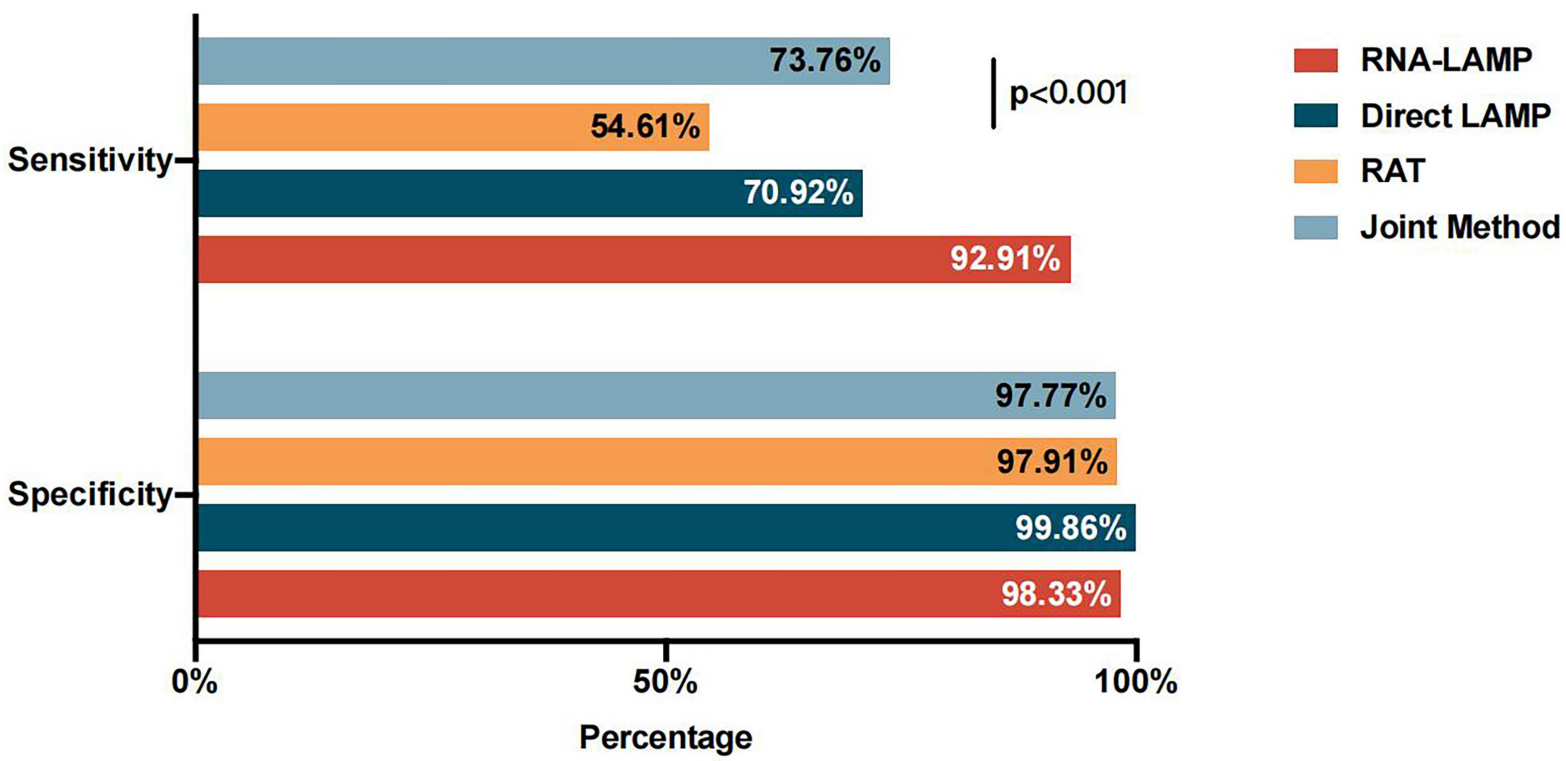
Diagnostic performance of the RNA-LAMP assay, direct LAMP assay, RAT, and joint method.

In addition, we further combined the application of the direct LAMP assay and the RAT (defined as the joint method) to improve the detection sensitivity. According to the practical application, if one of these tests yielded a positive result, the individual should be considered as a suspected case of COVID-19. Therefore, we analyzed the sensitivity and the specificity of the joint method and found that the sensitivity was 73.76% (95% CI, 65.56%–80.64%), significantly higher than that of the RAT alone (73.76% vs. 54.61%, *p* < 0.001) and slightly higher than that of the direct LAMP assay alone (73.76% vs. 70.92%, *p* = 0.125). The specificity of the joint method was also high (97.77%; Figure 5).

## Discussion

Several nucleic acid amplification approaches for diagnosis and monitoring of SARS-CoV-2 infections were developed in response to the COVID-19 pandemic. Molecular techniques, such as real-time PCR, are extremely sensitive and specific for detecting viral RNA and are currently recommended by the WHO for confirming diagnosis in symptomatic patients as well as for guiding public health decisions. However, local hospitals and clinics are bearing a heavy burden of detecting potential COVID-19 cases and treating patients. Therefore, integrated random-access, point-of-care molecular instruments for fast and accurate diagnosis of SARS-CoV-2 infections are urgently needed (Hou et al., 2020; Loeffelholz and Tang, 2021).

The data reported here describe the performance characteristics of the LAMP assay in detecting the nucleic acid of SARS-CoV-2, in both direct and RNA-extracted ways. We further performed a head-to-head comparison of the LAMP assay and RAT, considering practical use, and the results of this analysis included a relatively high sensitivity (direct LAMP, 70.92%; RNA-LAMP, 92.91%) and specificity (direct LAMP, 99.86%; RNA-LAMP, 98.33%) of the LAMP assay, especially using the extracted RNA, which was significantly better than the RAT in terms of sensitivity (54.61%), while the specificity remained similar (97.91%). According to a previous study, the direct LAMP assay was found to have a sensitivity of 67%, and the RNA-LAMP assay was reported to have a sensitivity of 97% in the detection of SARS-CoV-2, which are comparable to our results (Fowler et al., 2021). Furthermore, the direct LAMP assay was demonstrated to have a higher sensitivity than the RAT among both asymptomatic and symptomatic patients. The result approves the potential use of direct LAMP in rapid screening of both symptomatic and asymptomatic individuals, as previous studies have represented similar viral loads in asymptomatic and symptomatic patient groups (Arons et al., 2020; Kimball et al., 2020; Lavezzo et al., 2020; Cereda et al., 2021).

Although the RNA-LAMP assay showed a relatively higher sensitivity than the direct LAMP assay (92.91% vs. 70.92%, *p* < 0.001), RNA extraction is a significant rate-limiting step influencing COVID-19 diagnostic capacity. Consequently, despite a loss of sensitivity, the option to skip this step and test directly from a swab has significant benefits, including time and reagent savings and a more simplified methodology, reflecting the value of the application of the direct LAMP assay in the home environment or large-scale detection circumstances.

The performance of RATs for COVID-19 diagnosis has been intensively researched during the continuing COVID-19 pandemic (Khalid et al., 2022). In this head-to-head comparison between the direct LAMP assay and RAT, the LAMP assay demonstrated a higher sensitivity (70.92% vs. 54.61%, *p* < 0.001) and specificity (99.86% vs. 97.91%, *p* = 0.001) than the RAT. Earlier results are similar to the current findings: an *in vitro* experiment conducted by the Japanese government revealed that, compared to RT-PCR, RAT showed a sensitivity that varied from 50% to 66.7% (Nagura-Ikeda et al., 2020). A recent assessment of the literature, which included 16 trials, found that fast antigen testing for screening of asymptomatic persons can help reduce the spread of the disease (Loeffelholz and Tang, 2020; Walsh et al., 2022), suggesting the clinical application value of RATs. Considering that the direct LAMP assay has a higher sensitivity than RAT, we suppose that the LAMP assay could also play an important role in population screening. Furthermore, we combined the direct LAMP assay and RAT and found a sensitivity of 73.76%, higher than separate detecting by the LAMP assay or RAT. The result indicated the benefit of combining the two methods and providing a new application mode.

Besides, in this study, we demonstrated that the direct LAMP assay achieved a high positive rate (100%) in detection among patients with *C*_T_ values <28. However, among COVID-19 patients with *C*_T_ values of 30–35, the detection rate was relatively low (RNA-LAMP, 82.69%; direct LAMP, 30.77%). As RNA is a surrogate of live viral shedding, the results we found were in line with those of several previous studies, which reported that the virus cultivation was more viable when *C*_T_ values were < 25, while the positive rate declined obviously when the *C*_T_ values were > 30 (Bullard et al., 2020; Singanayagam et al., 2020; Sohn et al., 2020; Gniazdowski et al., 2021; Jaafar et al., 2021; Wölfl-Duchek et al., 2022). Previous publications have reported that it is unlikely that patients providing samples with high *C*_T_ values pose a high risk of transmission (La Scola et al., 2020). Combining the results, we found that in the early phase (≤7 days) since the first positive RT-PCR test, the direct LAMP assay performed well in both sensitivity and accuracy. We could arbitrarily infer that the LAMP assay could exhibit high efficacy in detecting initially infected individuals who are highly contagious.

This study has several limitations. First, this study was conducted in a single center, which resulted in a limited sample size. Thus, the results cannot truly reflect the sensitivity and specificity of the LAMP assay among the population. Second, the samples were not collected by the patients themselves, thus importing bias, considering that, when patients collect samples themselves, the specimen may not contain sufficient viral RNA since the sampling site may not be deep enough. The LAMP assay performance on samples might vary according to the level of detectable RNA (as a surrogate of live viral shedding). Therefore, the sensitivity and specificity could be lower when the LAMP assay is put into practical use. To further quantify the effects of other factors influencing test sensitivity, standardization of clinical accuracy studies and access to patient-level *C*_T_ values and duration of symptoms are needed.

Collectively, the LAMP assay showed relatively high sensitivity and specificity values and has a simple methodology with no requirement for additional equipment, requiring less time or expense than RT-PCR. Thus, we propose that the LAMP assay could complement the RT-PCR test when a higher sample throughput is required, especially in clinical settings with limited medical and human resources, because of its simplicity. Furthermore, the direct LAMP assay could be utilized as a point-of-care screening tool to quickly identify highly contagious individuals within a hospital or care unit or as a means for self-examination of home personnel during times of increased disease prevalence, and combining it with the RAT result could further increase the sensitivity. While ensuring that negative results should still be obtained through laboratory confirmation, LAMP could facilitate an immediate public health response, such as self-isolation or quarantine instructions, as well as rapid track-and-trace tools, benefiting surveillance programs aimed at limiting SARS-CoV-2 transmission in a community.

## Data availability statement

The raw data supporting the conclusions of this article will be made available by the authors, without undue reservation.

## Ethics statement

The studies involving human participants were reviewed and approved by Huashan Hospital Ethical Committee (2022-539). The patients/participants provided their written informed consent to participate in this study.

## Author contributions

GC, JC, HZ, JA, and SW participated in the experiments, and GC provided with technical supports. KL analyzed the data and wrote the draft of the paper. GC and JA polish the draft. WZ, MG, SW, and JA conceived the experiments. YY, QL, XZ, JC, YZ, ZF, JS, HW, and GY participated in enrolling the volunteers and collected the basic and clinical information of the subjects. All authors contributed to the article and approved the submitted version.

## Funding

This study was funded by research grants from the Shanghai Science and Technology Committee (20dz2210403, 20dz2260100, 20Z11901100, 21NL2600100, 20S31902700 and 21Y11900600), Shanghai Municipal Science and Technology Major Project (HS2021SHZX001), and National Natural Science Foundation of China (82041010).

## Conflict of interest

The authors declare that the research was conducted in the absence of any commercial or financial relationships that could be construed as a potential conflict of interest.

## Publisher’s note

All claims expressed in this article are solely those of the authors and do not necessarily represent those of their affiliated organizations, or those of the publisher, the editors and the reviewers. Any product that may be evaluated in this article, or claim that may be made by its manufacturer, is not guaranteed or endorsed by the publisher.
