# Supplementary material for: A diagnostic accuracy study comparing RNA LAMP, direct LAMP, and rapid antigen testing from nasopharyngeal swabs
Source: Front Microbiol. 2022 Dec 22;13:1063414. doi: 10.3389/fmicb.2022.1063414 (PMC9813509; doi:10.3389/fmicb.2022.1063414)
Supplement: Supplementary file 1 [file Data_Sheet_1.docx]

Supplementary Material

# Supplementary Figures

**Supplementary Figure 1. Enrollment flowchart.**


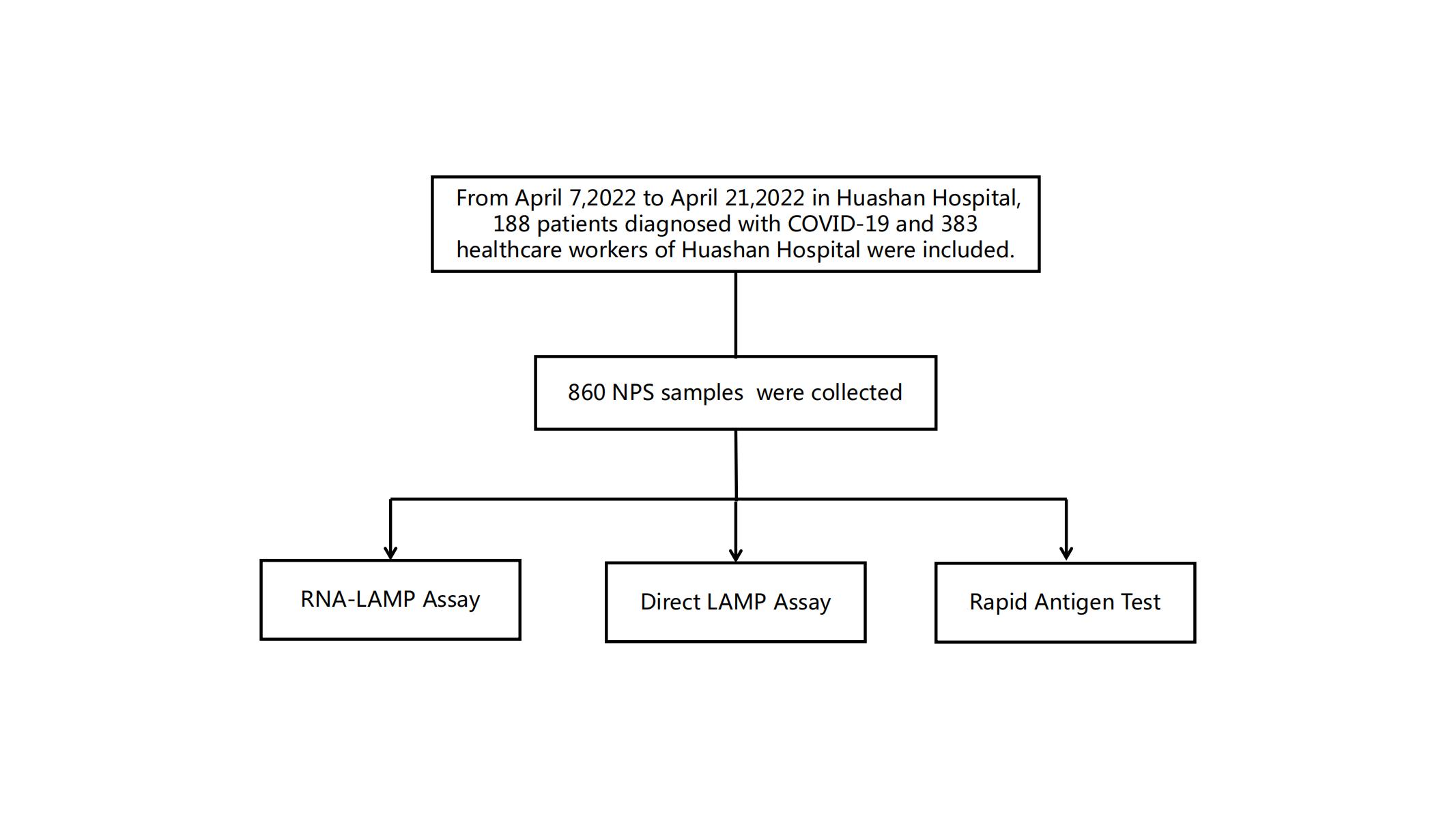


**Supplementary Figure 2. CT values of the specimens detected as negative or positive under different methods.**


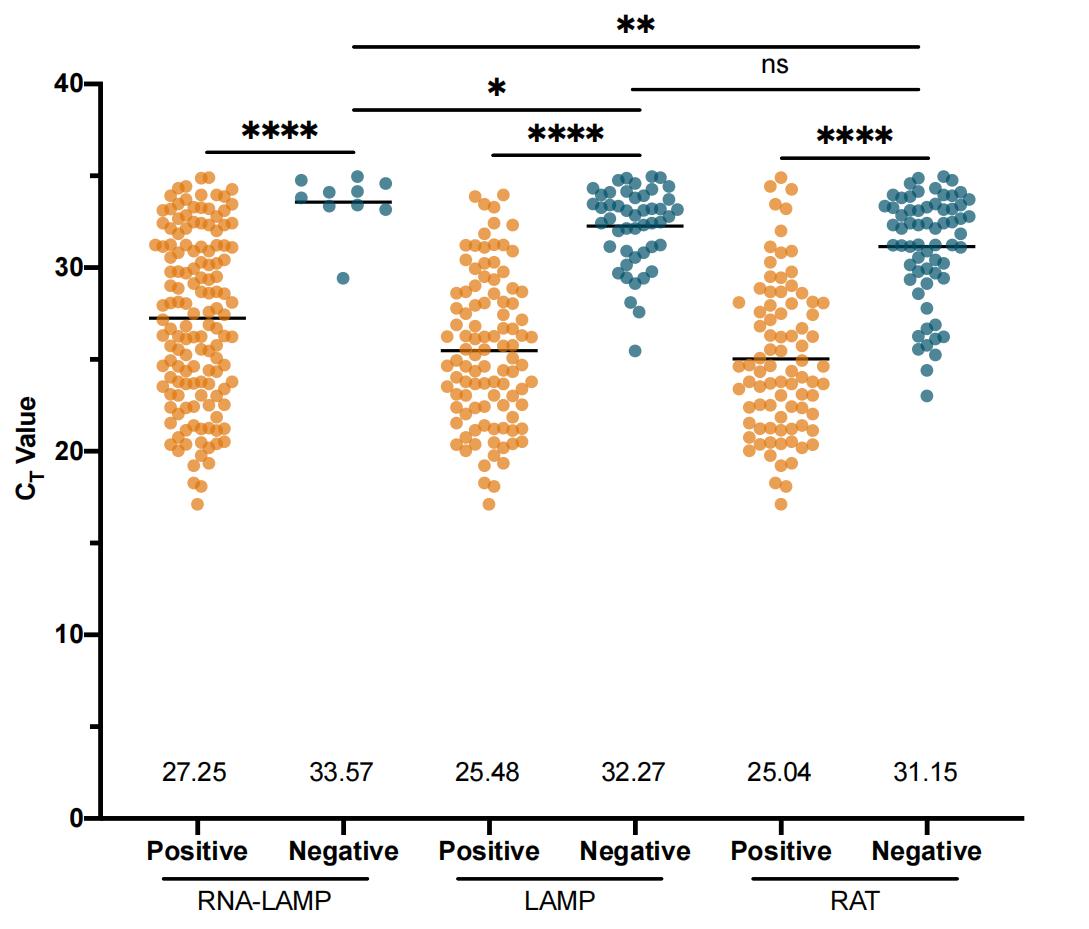


Yellow associates with the positive result. Green associates with the negative result.

# Supplementary Tables

**Supplementary Table 1. Enrollment and Exclusion Criteria**

| - **Enrollment Criteria** |
| --- |
| 1. Age $\geq$ 18 years 2. Patients in Huashan Hospital between April 7^th^, 2020 to April 21^st^, 2022 |
| 1. Healthcare workers in Huashan Hospital between April 7^th^, 2020 to April 21^st^, 2022 |
| 1. Agree to participate in the study |
| - **Exclusion Criteria** |
| 1. Age < 18 years |
| 1. Individuals unable to perform nasopharyngeal swab collection, such as post-operative nasopharyngeal, consciousness disorders |
| 1. Patients or healthcare workers who were unwilling to join the study |
|  |

**Supplementary Table 2. Performance of LAMP and RAT among asymptomatic and symptomatic patients**

| **Methods** | **Asymptomatic Patients (n=177)** | | **Symptomatic Patients (n=102)** | | **P value** |
| --- | --- | --- | --- | --- | --- |
|  | **Sensitivity (%) (95% CI)** | **Specificity (%) (95% CI)** | **Sensitivity (%) (95% CI)** | **Specificity (%) (95% CI)** |  |
| **RNA-LAMP** | 95.52 (86.62-98.84) | 93.58 (86.76-97.16) | 90.54 (80.91-95.79) | 96.24 (91.00-98.61) | p_4_ =0.825 |
| **Direct LAMP** | 71.64% (59.12-81.66) | 99.08 (94.26-99.95) | 70.27 (58.36-80.05) | 100.00 (96.50-100.00) | p_4_ =0.941 |
| **RAT** | 56.72 (44.08-68.58) | 92.66 (85.61-96.55) | 52.70 (40.83-64.29) | 94.74 (89.06-97.67) | p_4_ =0.796 |
| **p value** | p_1_<0.001, p_2_<0.001, p_3_=0.013 | p_1_=0.031, p_2_=1.000, p_3_=0.039 | p_1_<0.001, p_2_<0.001, p_3_=0.002 | p_1_=0.063, p_2_=0.754, p_3_=0.016 | / |

p_1_ stands for the significance of comparison between RNA-LAMP and direct LAMP

p_2_ stands for the significance of comparison between RNA-LAMP and RAT

p_3_ stands for the significance of comparison between direct LAMP and RAT

p_4_ stands for the significance of comparison of sensitivity between the asymptomatic and symptomatic patients
